# Empowerment of rural women. A bibliometric review

**DOI:** 10.12688/f1000research.166893.1

**Published:** 2025-07-15

**Authors:** Lindon Vela Meléndez, Juan Diego Dávila Cisneros, Elmer Llanos Díaz, Raquel Yovana Tello Flores, María del Pilar Fernández Celis, Lucinda Esperanza Castillo Seminario, Pedro Yesquén Zapata, Yefferson Llonto Caicedo

**Affiliations:** 1 Universidad Nacional Pedro Ruiz Gallo, Lambayeque, Lambayeque, 14013, Peru

**Keywords:** women's empowerment, rural women, bibliometrics, sustainable development

## Abstract

**Background:**

The empowerment of rural women is a key dimension for achieving gender equity and sustainable development. With this purpose, the present study analyzed the patterns and trends of scientific production on this topic using the Scopus database.

**Methodology:**

A quantitative approach was employed through bibliometric and scientometric techniques, using a collection of 376 documents (1983–2024). Through Bibliometrix (R) and VOSviewer, we identified co-authorship networks, thematic clusters, conceptual evolution, and indicators of productivity and impact.

**Results:**

Scientific production has shown sustained growth since 2015, driven by the Sustainable Development Goals (SDGs) and the increasing visibility of structural inequalities. The findings highlight institutional dispersion and limited international collaboration. Authors such as Mudhara M and Aziz N stand out for their recent impact. Semantic analysis reveals the centrality of terms such as agency, gender equality, and rural development. The structural thematic map shows that concepts like women’s rights and economic factors drive the field, while categories such as empowerment and leadership require further theoretical consolidation. Publication year spectroscopy reveals historical roots in the foundational works of Freire, Boserup, and Kabeer, who introduced emancipatory, relational, and critical perspectives on empowerment—now reinterpreted through intersectional approaches.

**Conclusions:**

The field has achieved conceptual maturity but still faces challenges related to methodological integration and global collaboration. It is recommended to broaden the analysis to other databases, strengthen scientific networks, and incorporate more integrative approaches into the contemporary rural agenda.

## Introduction

The empowerment of rural women is a central issue for the transformation of contemporary societies, as it directly contributes to achieving equity, reducing poverty, and promoting sustainable development. In recent decades, various institutions and international organizations have emphasized the need to highlight the structural conditions that perpetuate gender inequality in rural areas. Nevertheless, this agenda continues to face challenges related to local socioeconomic dynamics, patriarchal traditions, and digital divides that disproportionately affect rural women.

In this context, international scientific production has progressively incorporated intersectional approaches to examine how power structures impact the autonomy of rural women. The recognition of topics such as women’s leadership in agricultural communities, the integration of Information and Communication Technologies (ICT), and strategies for climate change resilience has given rise to a diverse body of research. However, this body of work remains fragmented, revealing the need for a systematizing perspective capable of identifying both the modes of knowledge production and the emerging trends that are shaping the development of academic literature on the empowerment of rural women.

In light of this reality, there is a noticeable lack of integrative studies that systematize the evolution of knowledge on the empowerment of rural women from a bibliometric perspective. While numerous empirical investigations exist, few explicitly explore how the scientific approach to this topic has developed within academic databases. Therefore, this article aims to analyze the patterns and trends in the scientific production on rural women’s empowerment in the Scopus database, with the goal of characterizing its historical evolution, addressed themes, collaboration networks, and existing research gaps in the field.

The guiding research question of this study is: What is the extent of scientific engagement with rural women’s empowerment in the history of publications indexed in Scopus?

This question seeks to uncover the dynamics of production, collaboration, and the thematic and semantic evolution that structure this field of study. As such, the following specific research questions are posed:

What has been the evolution of studies on rural empowerment factors?

Who are the most active authors in this area?

Which journals are most relevant to the topic?

From which fields of knowledge has the topic been investigated?

What levels of collaboration have authors engaged in?

Which institutions have acted as funders of research related to this phenomenon?

What is the semantic development surrounding the phenomenon under study?

What are the driving themes, perspectives, niches, and emerging topics in relation to the phenomenon?

What are the historical roots of the central concept or construct being examined?

The reviewed literature reveals diverse approaches to rural empowerment. Akhter et al. (2024) indicate that access to microcredit enhances women’s agency, though within a normative framework that constrains their structural autonomy. Bageant et al. (2024) point out methodological inconsistencies in empowerment indicators and emphasize the need for more context-sensitive metrics. Similarly, Udisha and Ambily (2024) highlight that digital literacy can serve as a catalyst for the economic inclusion of rural women, although access barriers persist. From a productive intervention perspective, Jiang et al. (2024) underscore the role of technical training in promoting female leadership, while Bitew et al. (2024) examine the factors influencing intrafamilial decision-making, where women continue to face deeply rooted cultural constraints.

In addition, recent research has emphasized the collective dimensions of empowerment. As demonstrated by Khatun et al. (2018), participation in community organizations fosters both self-worth and the public visibility of women.

In light of the breadth, complexity, and fragmentation of the field, this study is justified by its capacity to provide an overarching view of the scientific knowledge on rural women’s empowerment. By identifying key patterns of academic collaboration, the most active institutions and countries in the production of research on the topic, as well as emerging and consolidated themes, it becomes possible to detect thematic gaps and areas with potential for future investigation. In this regard, this critical analysis is essential to support both scientific production and public policy aimed at promoting gender equity in rural contexts.

## Methodology

The study adopted a quantitative approach and applied scientometric and bibliometric methods to analyze the scientific production related to the empowerment of rural women. This design is retrospective and descriptive, employs scientific mapping techniques, and seeks to define collaboration networks, thematic trends, and citation patterns. This type of analysis is aimed at describing and characterizing the structure and evolution of knowledge within a specific field.

To identify the final dataset, an initial search was conducted using key terms related to the research question, resulting in a total of 14,091 records. Inclusion criteria considered documents that contained the relevant keywords in both the title and abstract and were published up to and including the year 2024. As for exclusion criteria, articles that did not show a direct connection to the subject in the title or abstract were removed, as they were considered documentary noise, along with those from the year 2025, which were excluded due to the potential for incomplete records. Additionally, as part of the exclusion process, all documents whose titles, abstracts, or keywords included terms related to political, urban, or governmental approaches were automatically discarded through the application of the Boolean operator *NOT TITLE-ABS-KEY (politic OR urban OR government)**. This exclusion aimed to avoid thematic biases unrelated to the object of study and to ensure the conceptual relevance of the final sample.

**Figure 1.**
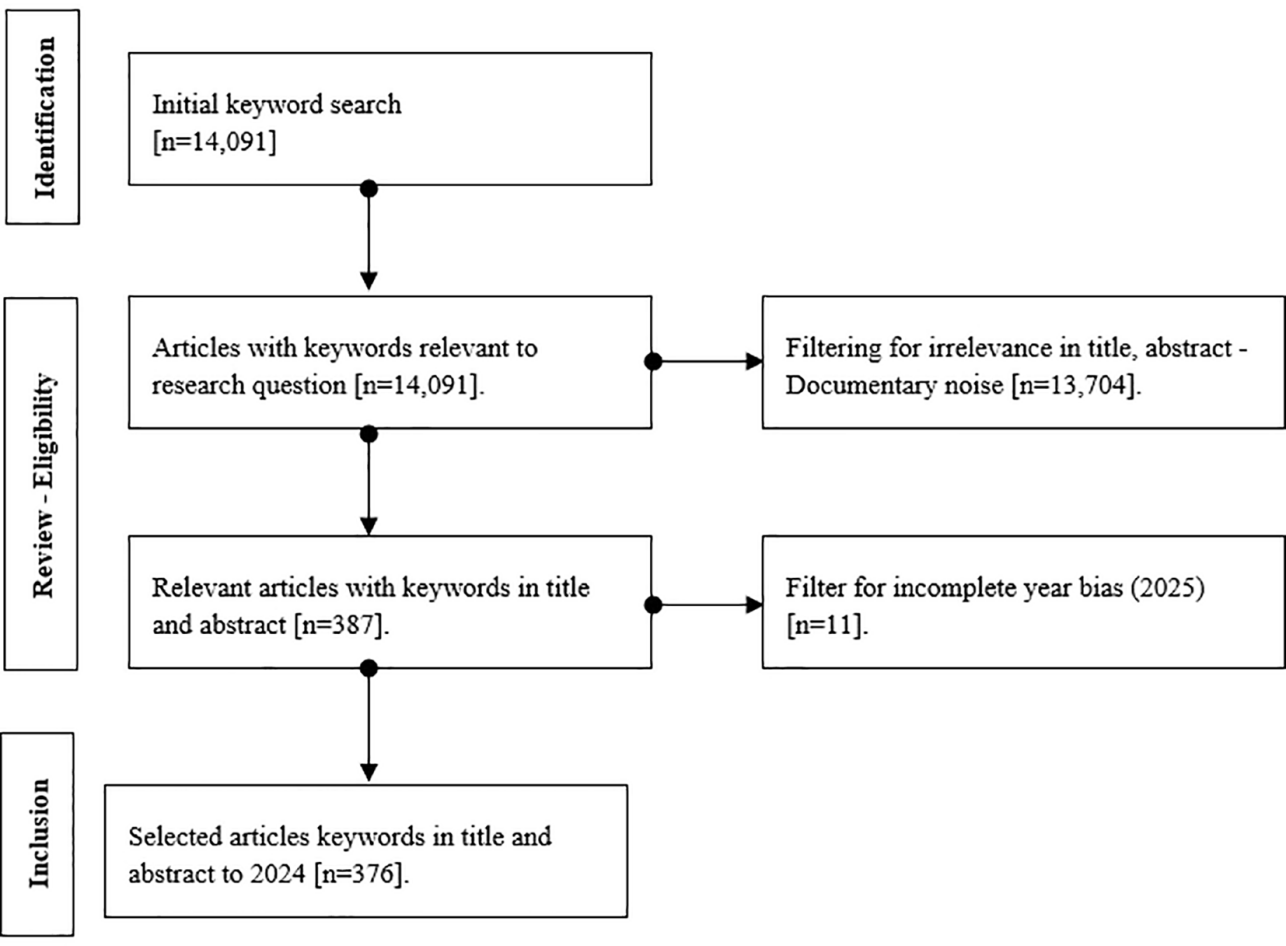
Document Collection Selection Process. Note: Graphical visualization of the systematic process used to identify the final dataset.

After applying these filters, a total of 376 articles were included for bibliometric analysis, as illustrated in Figure 1. The final search strategy was:

TITLE-ABS-KEY(“rural women” OR “rural woman” OR “peasant women” OR “female farmers” OR “agricultural women”) AND TITLE (empower OR “decision making” OR autonomy OR “gender equity” OR agency OR leadership) AND NOT TITLE-ABS-KEY (politic OR urban OR government).

This approach followed methodological precision guidelines for bibliometric reviews ( Donthu et al., 2021), and the data were exported in CSV format to facilitate processing.

The analysis was conducted using the Bibliometrix package in R, which enables the calculation of scientific productivity metrics, including the number of documents per year, the most influential authors and institutions, and impact indicators such as the h-index and g-index ( Aria & Cuccurullo, 2017). To visualize collaboration networks and co-word associations, VOSviewer was employed; this application allows for the implementation of clustering algorithms and normalization by association strength to identify thematic clusters and co-authorship groups ( Van Eck & Waltman, 2010). The figures generated were refined using Datawrapper to enhance their visual clarity.

## Results and discussion

In the bibliometric review on rural women’s empowerment, a total of 376 documents published between 1983 and 2024 were analyzed, originating from 285 sources. The study reveals an annual growth rate of 6.49%, indicating that scientific interest in the topic dates back over four decades. The average number of citations per document is 15.83, reflecting a moderate impact. The average document age is 8.59 years, suggesting the typical time span during which publications continue to generate interest in the scientific community.

The thematic content is diverse, with 952 keywords derived from Keywords Plus (ID) and 846 provided by the authors themselves (DE), highlighting the conceptual richness of the field. In terms of authorship, 979 researchers contributed to the publications, with single-author works being relatively scarce (90 documents). The average number of co-authors per document is 2.74, and 18.35% of the works involve international collaboration, reflecting a gradual openness toward global academic networks.

Overall, the indicators point to a growing field, characterized by increasing collaborative articulation and a plural thematic foundation—factors that underscore the academic relevance of rural women’s empowerment, as summarized in Table 1.

**Table 1.** Main bibliometric indicators of the collection.

Description	Results
**Main information about data**	
Timespan	1983:2024
Sources (Journals, Books, etc.)	285
Documents	376
Annual growth rate %	6.49
Document average age	8.59
Average citations per doc	15.83
References	13513
**Document contents**	
Keywords plus (ID)	952
Author’s keywords (DE)	846
**Authors**	
Authors	979
Authors of single-authored docs	88
**Authors collaboration**	
Single-authored docs	90
Co-authors per doc	2.74
International co-authorships %	18.35

Note: Bibliometric indicators elaborated with Bibliometrix.

**Figure 2.**
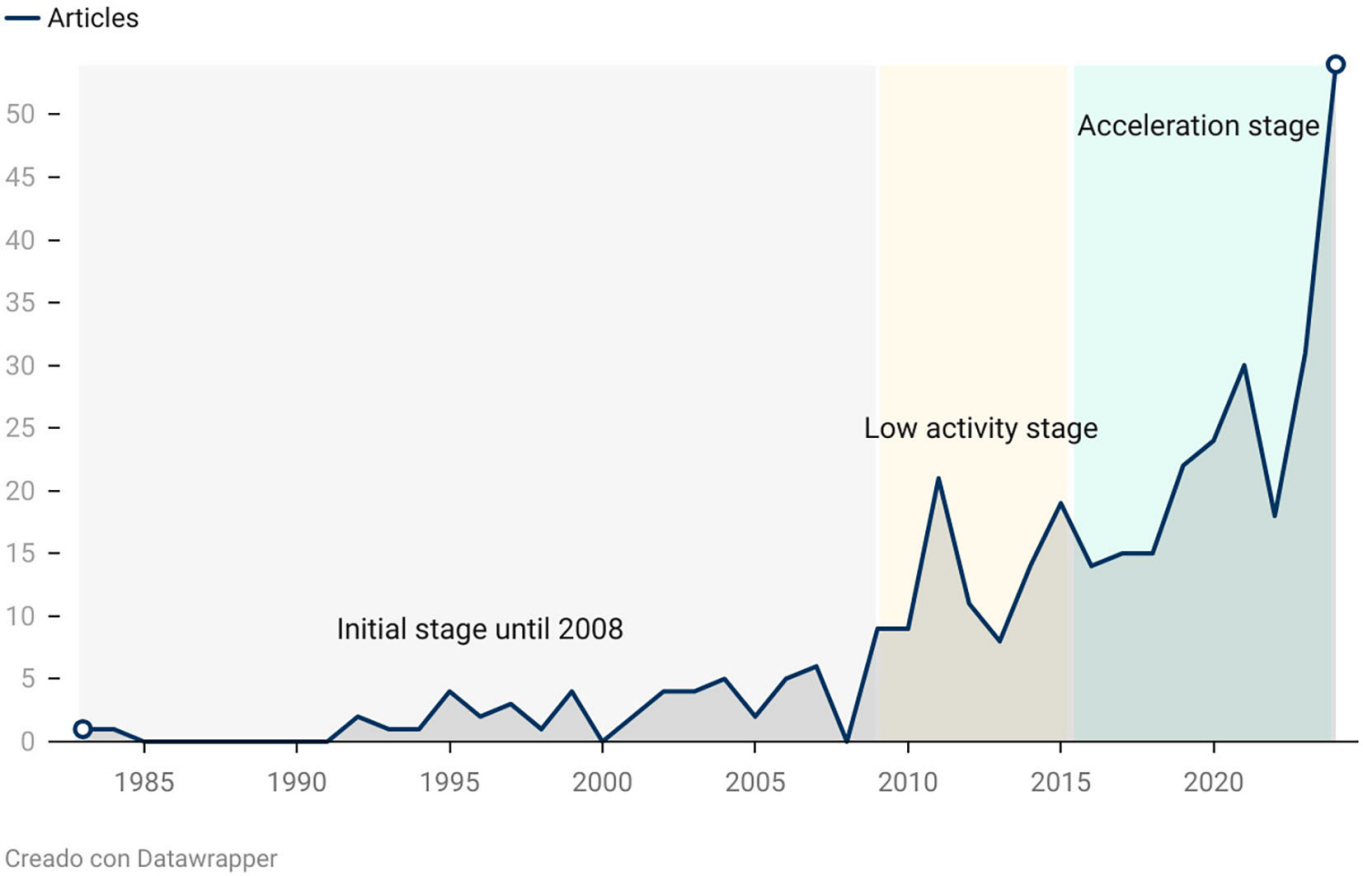
Evolution of publications on rural women's empowerment. Note: Processed in Bibliometrix based on Scopus metadata, visual presentation with Datawrapper.

The evolution of scientific production on rural women’s empowerment can be divided into three stages: an initial phase of low visibility (up to 2008), a period of low activity (2009–2014), and sustained acceleration beginning in 2015, as illustrated in Figure 2.

In the initial phase, the number of publications was limited, reflecting a development approach centered on aggregated economic indicators and a limited consideration of women’s agency. As Sen (2001) points out, development entails expanding freedoms, not just increasing income; however, rural women were often viewed more as beneficiaries than as agents of change. Kabeer (1999) reinforces this view by defining empowerment as the ability to make strategic life choices in contexts where this capacity was previously denied—a dimension largely absent in the literature of that period.

The low-activity stage coincides with the 2008 financial crisis, which redirected funding priorities toward sectors such as science and technology. Vives (2010) notes that financial regulation and economic recovery dominated the agenda, pushing social issues such as gender equity in rural settings to the margins.

Since 2015, scientific output on the topic has experienced sustained growth, driven by the inclusion of rural women’s empowerment in the Sustainable Development Goals (particularly SDG 5) and by the COVID-19 pandemic, which exposed the structural inequalities faced by rural women. Studies such as Moreno et al. (2021) demonstrate that programs like Emprendiendo una Vida Mejor in Honduras not only improved economic security but also enhanced participants’ self-esteem, agency, and decision-making capacity—evidence of a more comprehensive approach to empowerment.

The findings of this study, which reveal a significant increase in scientific production on rural women’s empowerment starting in 2015, align with those of Jiang et al. (2024), who emphasize the growing recognition of women’s empowerment in sustainable rural revitalization programs. Their study shows how initiatives focused on strengthening traditional skills—such as bamboo weaving—have gained visibility in the past decade, mirroring the global trend observed in our publication curve. This convergence highlights how the current sociopolitical context, particularly the integration of empowerment into the Sustainable Development Goals, has catalyzed intensified academic efforts to bring attention to female agency in rural environments.

Author productivity is measured using indicators such as the h-index and m-index, which allow for the identification of both productivity and the relative impact of authors over time, as shown in Table 2. The h-index reflects an author’s productivity and the number of citations received, while the m-index adjusts this measure based on the number of years of active publication, making it particularly useful for comparing shorter and longer academic trajectories under equal conditions ( García-Villar & García-Santos, 2021).

**Table 2.** Author productivity.

Author	Index h	Index m	Quotes	Publications	Year of start of publications
MUDHARA M	3	0,300	108	3	2016
ANDREW TN	2	0,105	14	2	2007
AZIZ N	2	0,333	97	2	2020
BOGALE A	2	0,200	98	2	2016
DATTA S	2	0,400	47	2	2021
FONTANA M	2	0,286	56	2	2019
HAQUE MA	2	0,500	7	2	2022
ISHFAQ S	2	0,500	10	3	2022
JEJEEBHOY SJ	2	0,143	58	2	2012
JOSEPH MK	2	0,105	14	2	2007

Note: Processed in Bibliometrix based on Scopus metadata.

These concepts and indicators reveal that Mudhara M stands out as one of the most influential authors, with an h-index of 3 and a total of 108 citations across three publications since 2016. Although his m-index (0.300) is not the highest, his cumulative impact is significant. In contrast, authors such as Haque MA and Ishfaq S have the highest m-index in the dataset (0.500), reflecting remarkable impact in a short period, as they began publishing in 2022. This phenomenon suggests a growing interest and citation rate for recent research on the topic. Other authors, such as Datta S (m-index = 0.400, 47 citations since 2021) and Aziz N (m-index = 0.333, 97 citations since 2020), also show upward trajectories. Meanwhile, figures such as Joseph MK and Andrew TN, who began publishing in 2007, display a lower m-index (0.105), indicating relatively limited long-term impact.

This bibliometric behavior highlights a generational shift in the scientific authorship on rural women’s empowerment, with emerging researchers achieving high impact in short timeframes. This trend aligns with recent observations on the utility of the m-index for detecting rapidly growing, emerging research areas, thus complementing the limitations of the traditional h-index ( Seoane-Mato et al., 2025).

**Figure 3.**
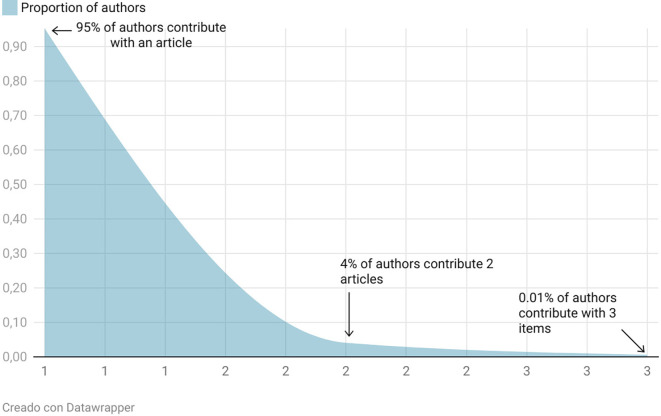
Lotka’s law. Note: Processed in Bibliometrix based on Scopus metadata, visual presentation with Datawrapper.

In the analysis of scientific productivity on rural women’s empowerment, a highly unequal distribution of authorship is observed, consistent with Lotka’s Law, one of the classical laws of bibliometrics, as depicted in Figure 3. According to this law, the majority of researchers contribute only a single publication, while only a small minority produce multiple works. In this case, 95% of authors contributed with just one article, 4% with two articles, and only 0.01% have published three articles. This asymmetry reflects a strong concentration of output among a few authors, while a broad base of researchers contribute only occasionally.

This phenomenon is characteristic of knowledge generation dynamics in applied social sciences and has been recently documented in bibliometric studies such as García-Villar and García-Santos (2021), who emphasize that “Lotka’s Law helps identify the degree of concentration in authorship, serving as a tool for detecting collaboration structures and specialization within a field.” This pattern suggests that while there is growing interest among new researchers, only a few maintain continuous output—likely reflecting both thematic specialization and structural limitations to conducting research in this area.

**Figure 4.**
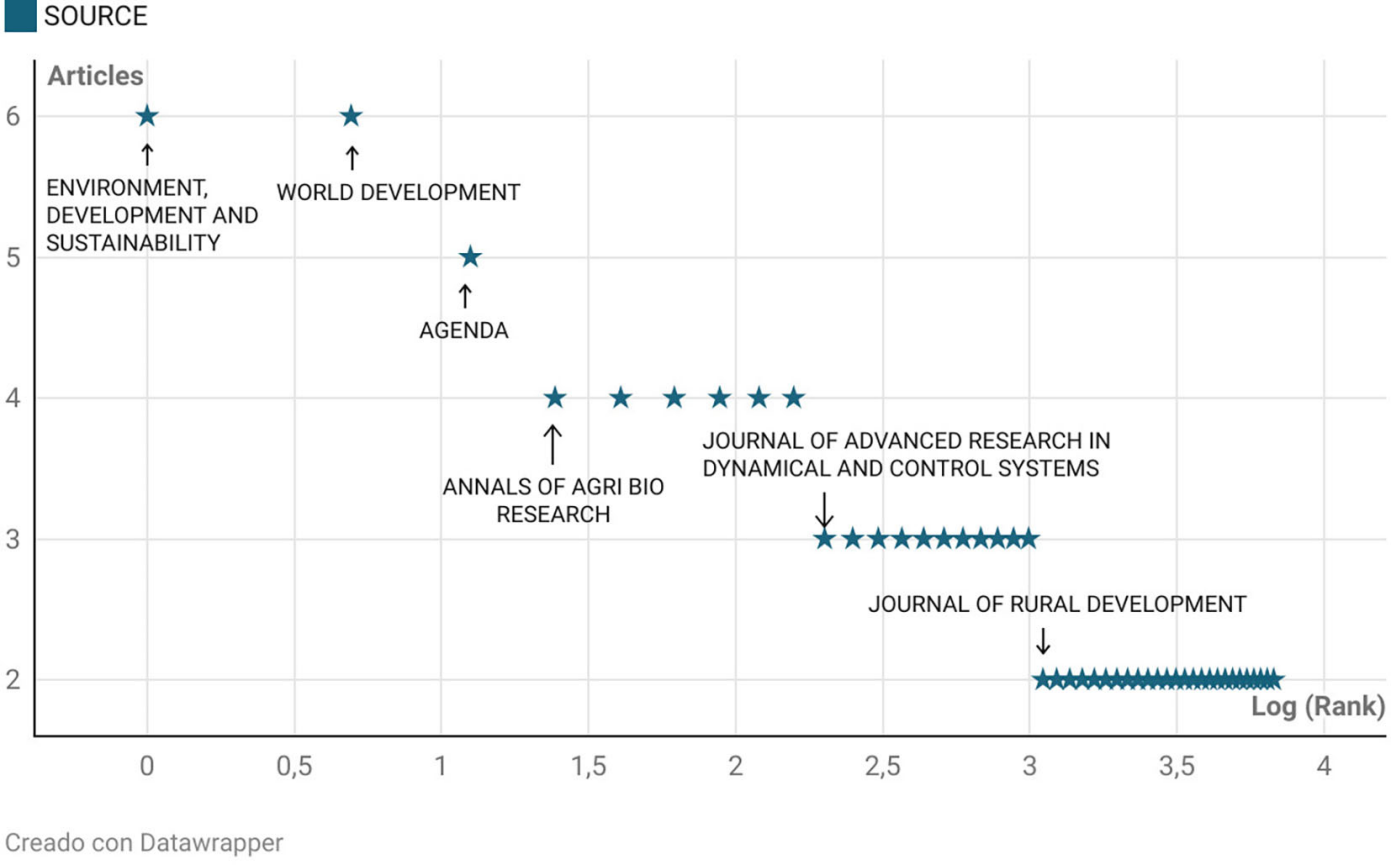
Bradford’s law. Note: Processed in Bibliometrix based on Scopus metadata, visual presentation with Datawrapper.

Figure 4 represents an application of Bradford’s Law to the analysis of scientific journals publishing on rural women’s empowerment. This law, one of the foundational principles of bibliometrics, states that a small number of journals concentrate the majority of relevant articles on a specific topic, forming what is known as the core zone or Zone 1.

In the present study, the journals Environment, Development and Sustainability, World Development, and Agenda stand out as the Bradford core, each accumulating more than five publications on the subject. These journals serve as the primary channels for scientific dissemination, fulfilling the concentration principle originally described by Bradford. As the logarithm of the rank increases, a progressive dispersion of articles is observed toward journals with lower relative frequency, such as Journal of Rural Development or Annals of Agri Bio Research, confirming the typical dispersion pattern described by this law.

This behavior has been validated in recent studies, such as that by Fraga-Graells et al. (2022), who applied Bradford’s Law in their bibliometric analysis of refractive surgery and found that four journals concentrated the majority of publications—thus confirming the concept of an informational core. Similarly, Xue and Liu (2024) emphasize that the temporal evolution of Bradford curves continues to be a useful tool for guiding scientific literature acquisition in specialized libraries, particularly when identifying high-productivity journals in an emerging field.

**Figure 5.**
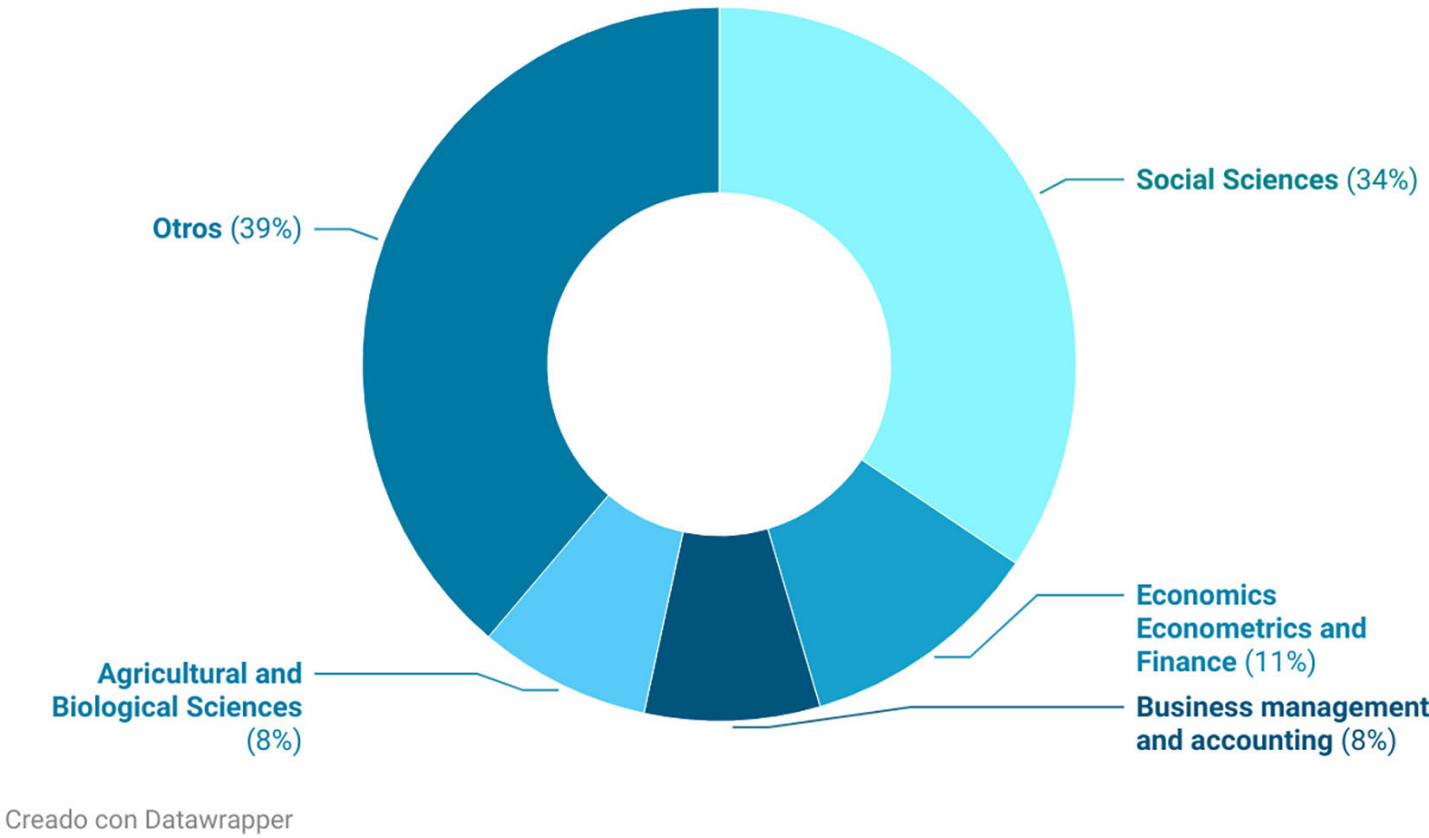
Areas of knowledge. Note: Processed in Bibliometrix based on Scopus metadata, visual presentation with Datawrapper.

Figure 5 illustrates the distribution of knowledge areas contributing to research on rural women’s empowerment. Social sciences stand out prominently (34%), which aligns with the multidimensional nature of empowerment, encompassing sociocultural, political, and gender-related dimensions. This is followed by economics, econometrics, and finance (11%) and business, management, and accounting (8%), indicating a growing focus on economic autonomy as a key component of empowerment.

Agricultural and biological sciences account for 8%, highlighting the relevance of rural and productive contexts in the research. Finally, 39% of the studies are grouped under Other, revealing a dispersion of topics and the participation of various disciplines—likely including public health, education, and environmental development.

This distribution underscores the interdisciplinary character of the field while also confirming the central role of social sciences as the primary analytical framework. This pattern is consistent with the approach used by Akhter et al. (2024), who analyze empowerment through the interaction between social structures and financial decision-making. Similarly, authors such as Udisha and Ambily (2024) incorporate technological and social variables to reinforce the multidimensional nature of empowerment and the importance of interdisciplinary approaches.

**Figure 6.**
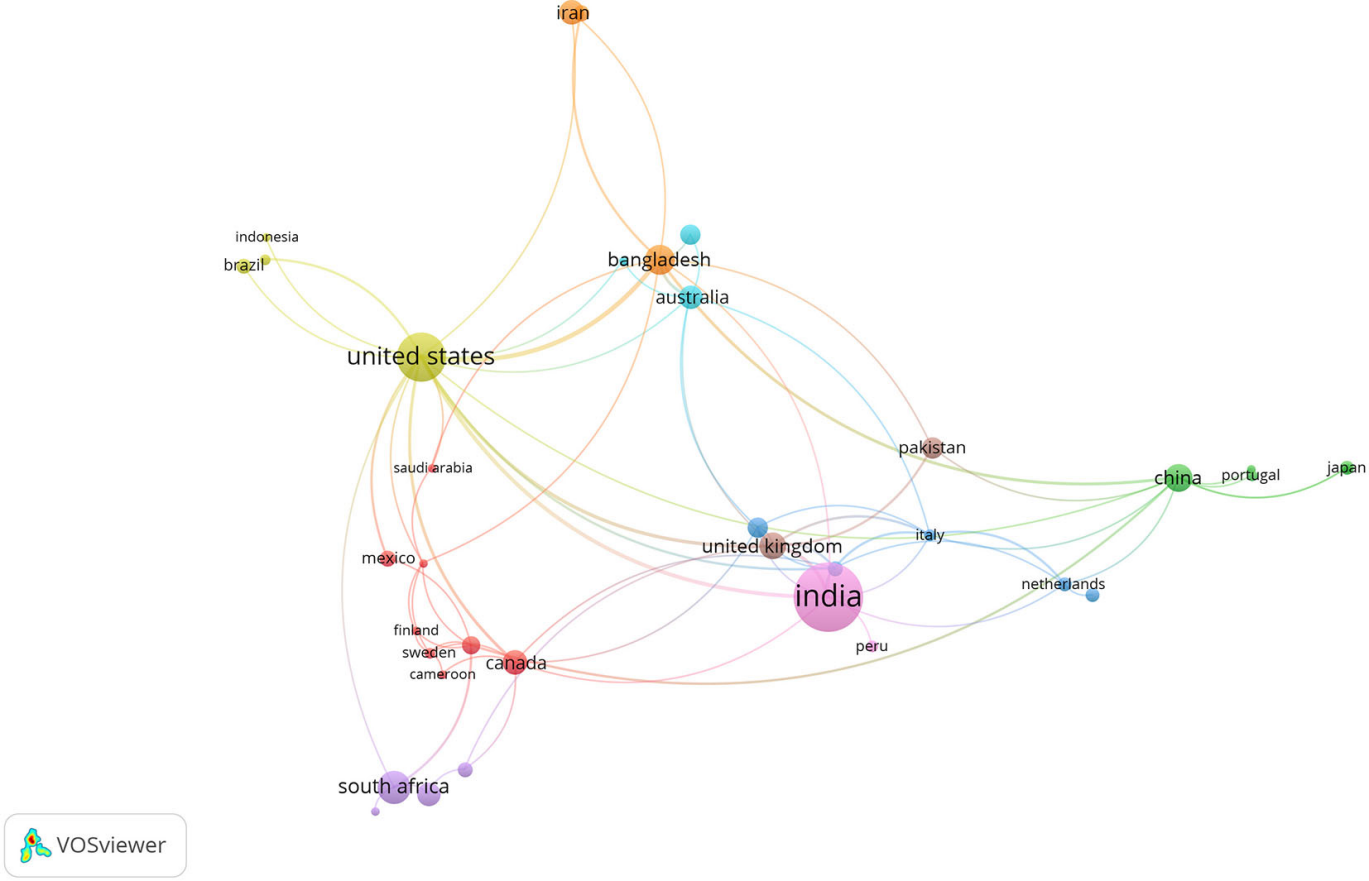
Author collaboration network. Note: Processed in VOSViewer based on Scopus metadata.

Figure 6 presents a co-authorship network map in research on rural women’s empowerment. Each node represents an author, and the links indicate collaboration in publications. Multiple disconnected clusters are observed, suggesting a high degree of fragmentation within the scientific community, with groups of authors working in isolation.

Some visible clusters—such as those led by Lentz, Erin and Fontana, Marzia (red), or Ishfaq, Sidra and Kauser, Shahzad (green)—indicate cores of active collaboration. However, most authors appear as isolated nodes or with very few links, reflecting limited international or interdisciplinary cooperation.

This low density of connections is characteristic of emerging or specialized fields where broad scientific collaboration networks have yet to consolidate.

The divergence between thematic diversity and author fragmentation suggests that interdisciplinarity is operating primarily at the conceptual level, rather than through collaborative authorship. This highlights the need to strengthen research networks that can support more integrated, sustained, and global approaches.

**Figure 7.**
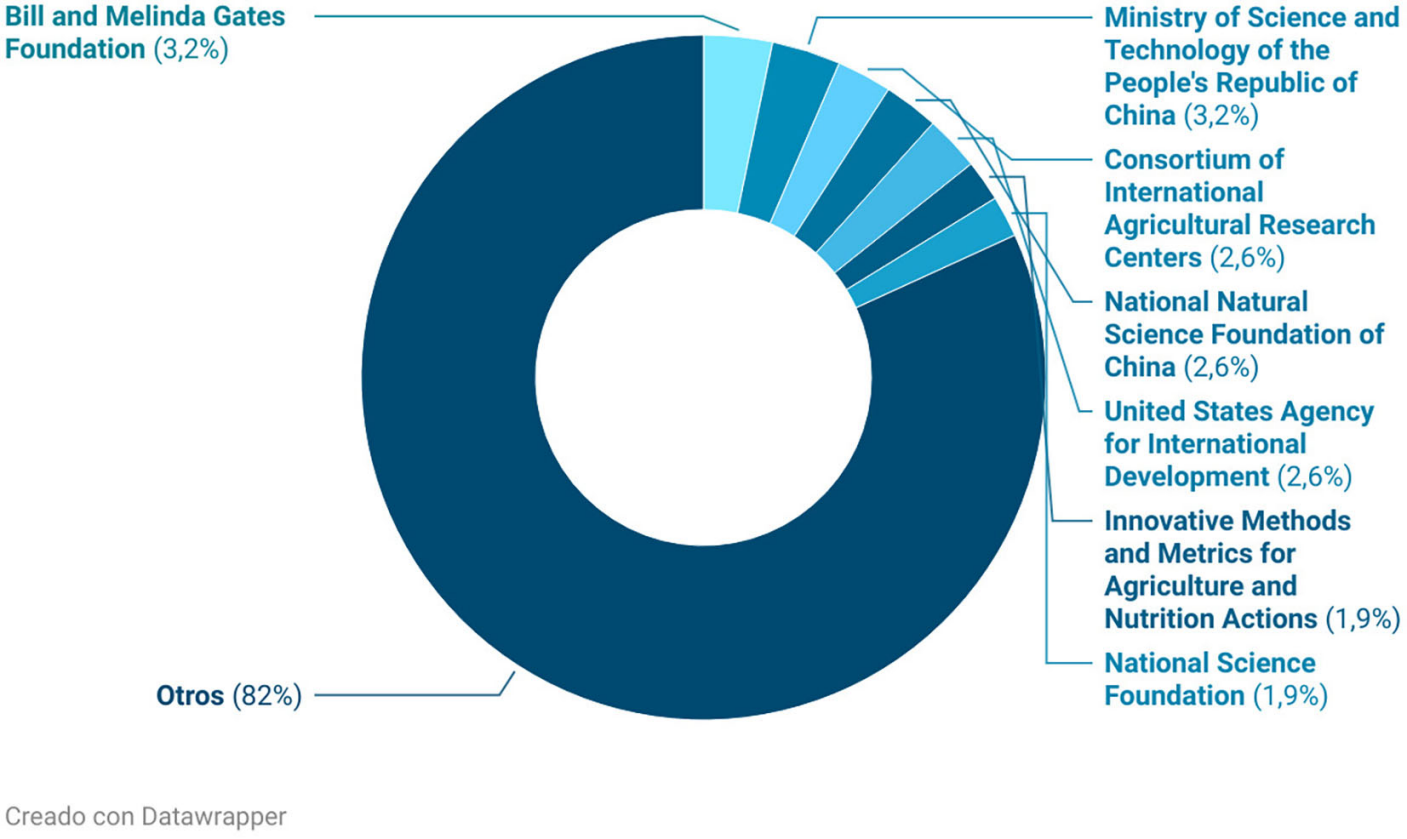
Sources of research funding. Note: Processed in Bibliometrix based on Scopus metadata, visual presentation with Datawrapper.

Figure 7 illustrates the institutional distribution of funding for research on rural women’s empowerment, providing key insights from a scientometric and bibliometric perspective. The most notable finding is that 82% of funding originates from entities grouped under the category “Others,” suggesting a high level of institutional dispersion—likely reflecting the predominance of local sources, universities, NGOs, and agencies with relatively low investment capacity. This fragmentation highlights the decentralized nature of the field while also exposing the lack of consolidated large-scale funding structures.

Among the clearly identified funders, the Bill and Melinda Gates Foundation (3.2%) and the Ministry of Science and Technology of the People’s Republic of China (3.2%) stand out, both institutions with strong commitments to equity, sustainable agriculture, and food security. Also present are multilateral and international agencies such as USAID, CGIAR (Consortium of International Agricultural Research Centers), and the U.S. National Science Foundation. The involvement of these organizations reinforces the notion that rural women’s empowerment has been increasingly recognized as a relevant issue on the global development agenda.

Moreover, the prominent participation of Chinese foundations—such as the National Natural Science Foundation of China—points to China’s growing interest in leading South–South cooperation initiatives, particularly on themes related to rural development and sustainability.

From a bibliometric standpoint, this distribution reveals three important trends. First, it reflects a partial internationalization of the field, with some high-profile funders but no dominant core, which may promote thematic diversity while limiting the establishment of global research standards. Second, it confirms the multisectoral nature of the topic, with contributions from international cooperation, philanthropy, agricultural science, and the social sciences. Third, it underscores a strategic opportunity to foster stronger institutional networks and international collaboration, which could enhance scientific capacity in this area.

Taken together, these data indicate that research on rural women’s empowerment is still in a phase of structural consolidation, with a thematically mature but institutionally fragmented field.

**Figure 8.**
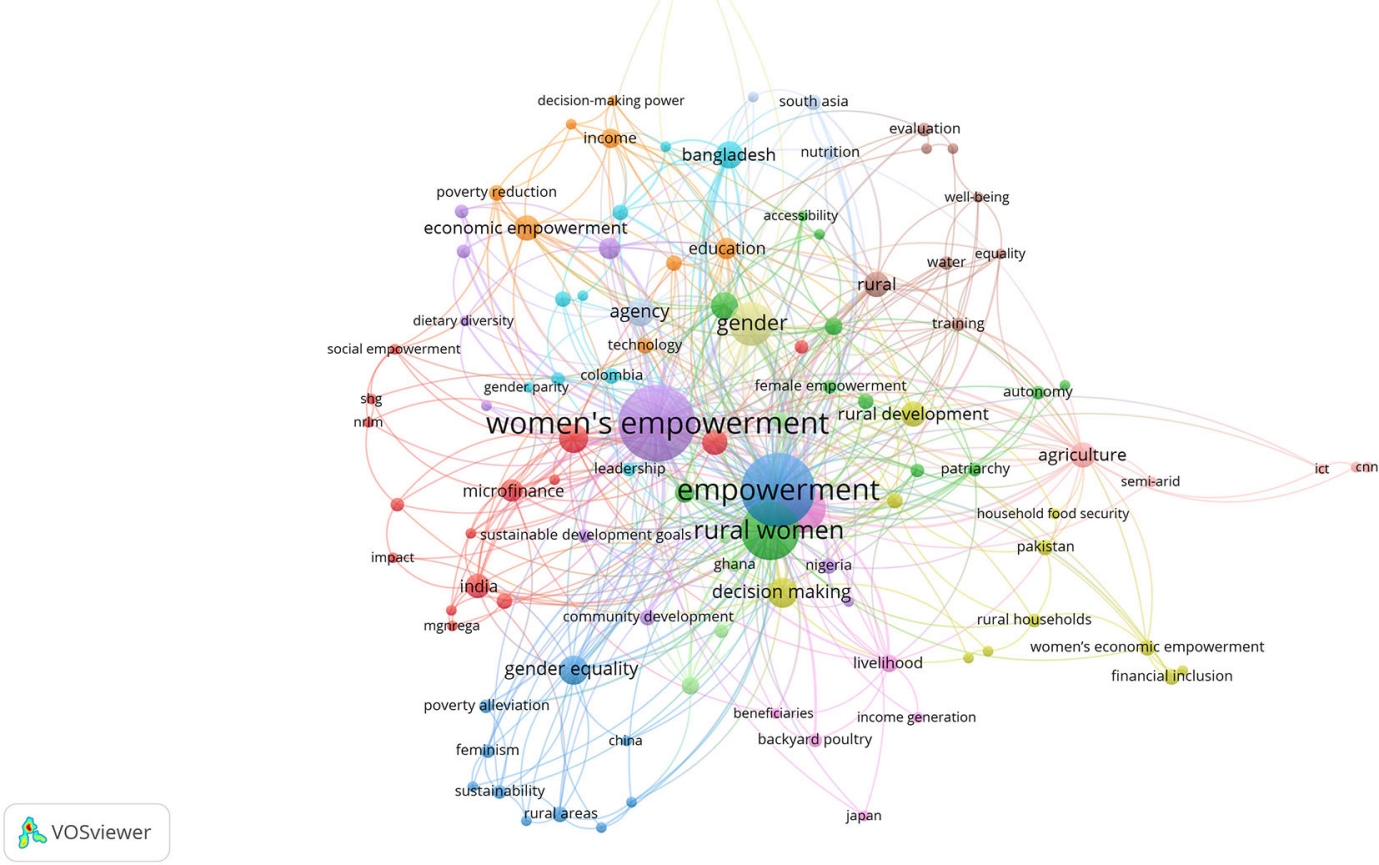
Semantic map on rural women's empowerment. Note: Processed in VOSViewer based on Scopus metadata.

Figure 8, the semantic map, clearly illustrates that rural women’s empowerment is a profoundly multidimensional concept, interweaving terms such as agency, decision making, economic empowerment, gender equality, and rural development. At the core of the discourse on this topic are ideas linked to the capacity to make decisions, access resources, and transform traditional environments. This suggests an understanding of empowerment not merely as material access, but as a process of personal and collective transformation.

Surrounding this thematic nucleus are various lines of research. Some focus on economic aspects, such as microfinance, food security, and livelihoods; others on symbolic and normative dimensions, including leadership, gender equality, and education. The diversity shown in this map reflects that empowerment is not an isolated process—it interacts across multiple layers: from household to politics, from land to institutions.

However, this richness also reveals a degree of fragmentation: many topics coexist but do not necessarily intersect or engage in dialogue. As Moreno et al. (2021) point out, the most effective programs are those that simultaneously integrate access, agency, and social transformation, rather than addressing these dimensions separately. Similarly, Aguirre (2014) highlights that rural women are not only empowered through access to resources but by redefining their place in the world based on their own experiences and traditions.

Ultimately, this map reflects not only a broad academic field but also a pressing challenge: moving from studies that isolate empowerment dimensions to truly integrative approaches—ones that recognize the complexity and transformative potential of women’s leadership in rural contexts.

**Figure 9.**
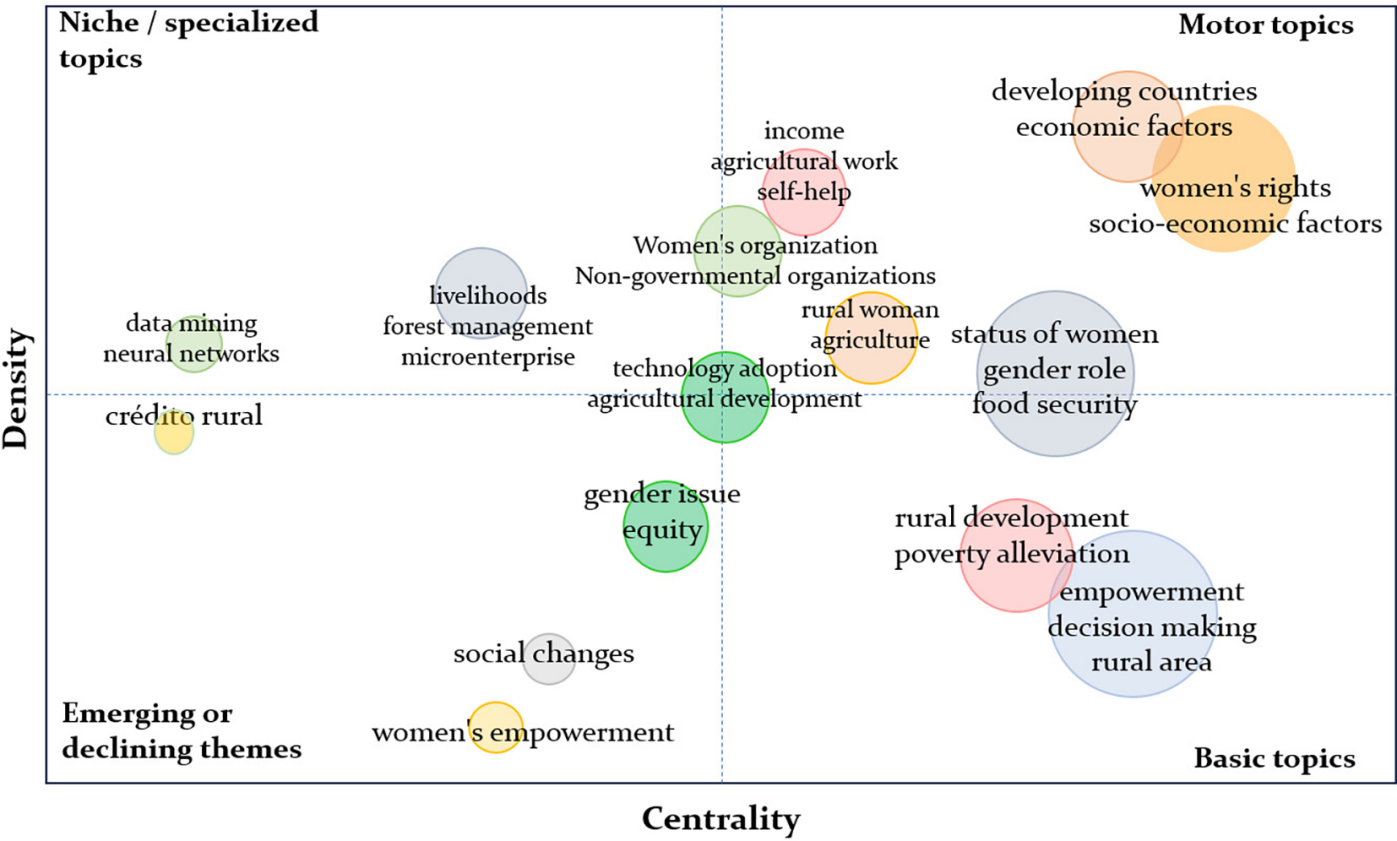
Thematic map. Note: Processed in Bibliometrix based on Scopus metadata.

The structural thematic map generated with Bibliometrix offers a clear view of the conceptual configuration of the field of rural women’s empowerment. Using the dimensions of centrality (the thematic importance within the network) and density (the degree of internal development), it is possible to distinguish between the consolidated core and emerging margins of the field, as illustrated in Figure 9.

In the motor themes quadrant, prominent concepts include women’s rights, economic factors, and developing countries, which function as well-developed and highly transversal organizing axes. These themes sustain a global and multiscalar agenda that connects gender equity with socioeconomic justice and sustainable development. The centrality of these axes aligns with the multidimensional empowerment approach highlighted in recent studies such as Moreno et al. (2021), which demonstrate how access to resources, agency, and symbolic transformation operate jointly to foster real autonomy in vulnerable rural contexts.

In the basic themes’ quadrant, core concepts such as empowerment, rural development, and decision-making appear. While frequently used, these themes exhibit lower density, suggesting that they still require theoretical and operational consolidation. As noted by Lorenzen et al. (2023), the livelihood strategies of rural women—particularly in the post-pandemic context—highlight the need to reformulate classical approaches and adopt frameworks more sensitive to gender and territory.

Niche themes, such as microenterprise, forest management, and livelihoods, demonstrate high density but low centrality. This indicates advanced thematic development, yet with limited integration into the core of the field. These specialized areas represent opportunities for interdisciplinary articulation, particularly if linked to gender and sustainability agendas, as explored in gender-focused rural extension initiatives in Brazil ( Holgado-Silva et al., 2023).

Finally, the emerging or declining themes quadrant includes concepts like women’s empowerment and social change, whose low centrality and density may signal an ongoing reformulation rather than abandonment. This is consistent with recent trends that seek to deconstruct empowerment into specific dimensions—economic, digital, and symbolic—as discussed by Vega-Barrios et al. (2025) in their analysis of academic women and technology.

Taken together, the thematic map reveals a field in transition: with solid cores, areas in need of consolidation, and a periphery offering space for innovation and theoretical convergence. These conditions invite a rearticulation of empowerment through frameworks that integrate gender, territory, labor, agency, and public policy.

**Figure 10.**
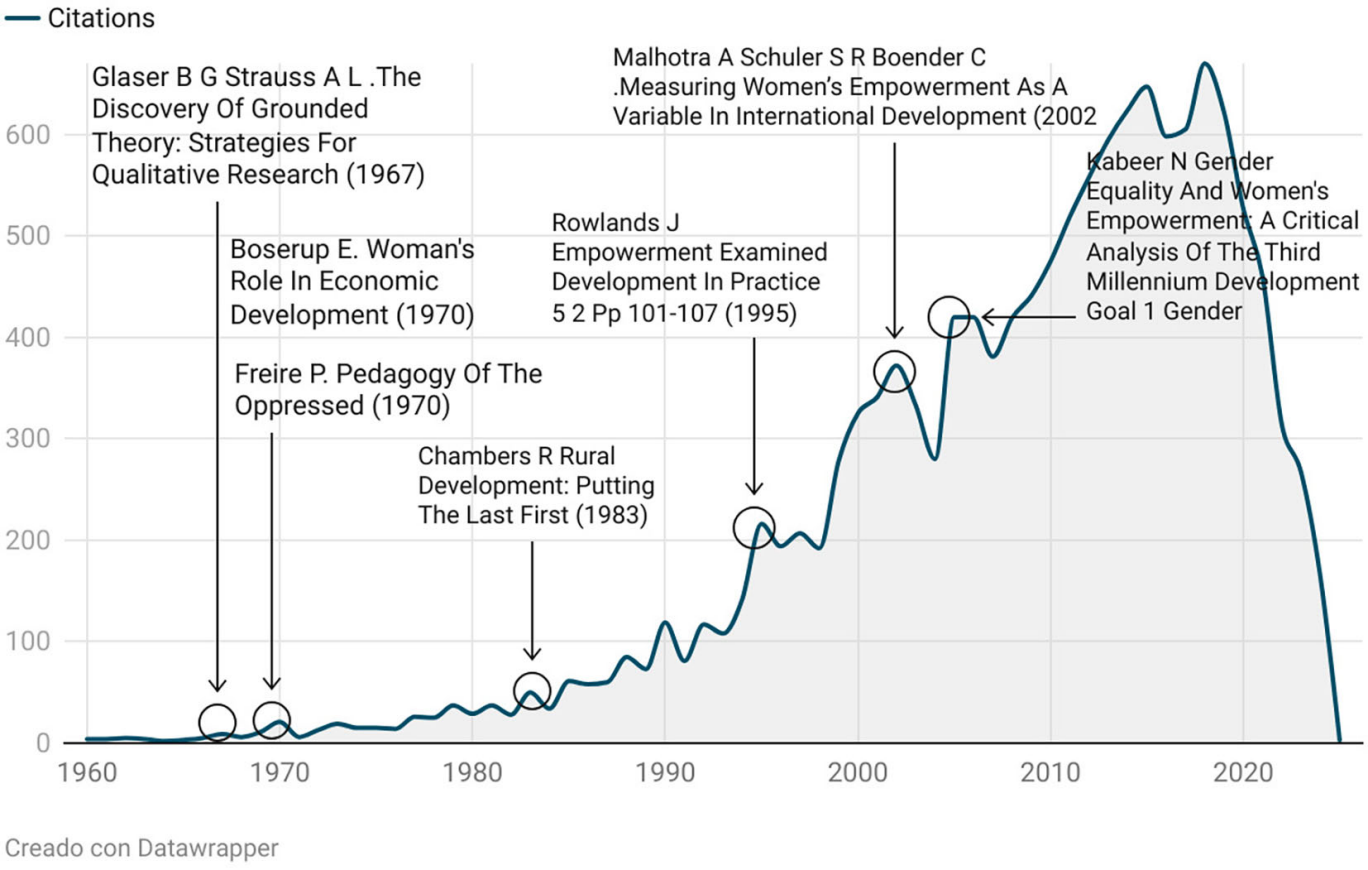
Historical roots of the empowerment concept. Note: Processed in Bibliometrix based on Scopus metadata, visual presentation with Datawrapper.

The concept of rural women’s empowerment has followed a complex and multidimensional trajectory, whose roots can be clearly identified through the Reference Publication Year Spectroscopy (RPYS) technique, as shown in Figure 10. This bibliometric tool enables the tracing of the most highly cited seminal texts that have shaped the field, revealing an evolution that spans from critical development frameworks to participatory approaches and measurement methodologies.

### Foundational Roots: Development Critique and Emancipation (1967–1983)

The first significant peak corresponds to Glaser and Strauss’s The Discovery of Grounded Theory ( 1967), a foundational work that introduced grounded theory methodology, enabling the study of empowerment from the lived experiences of social actors. Simultaneously, Paulo Freire (1970), in Pedagogy of the Oppressed, introduced the concept of conscientização (critical consciousness), defined as the capacity of the oppressed to read and transform their reality—an essential philosophical foundation for the idea of emancipatory empowerment.

That same year, Ester Boserup (1970) published Woman’s Role in Economic Development, the first work to demonstrate how economic development systematically marginalized rural women, paving the way for the Women in Development (WID) approach. Later, Robert Chambers (1983), in Rural Development: Putting the Last First, expanded the critique of top-down development models by proposing participatory methodologies that recognize local knowledge and promote community agency.

### Conceptual Formulation: Power, Agency, and Social Relations (1995)

In the 1990s, the concept of empowerment began to take clearer shape. In her influential article Empowerment Examined, Rowlands (1995) proposed a typology of power—power over, power to, power with, and power within—which allowed for the analysis of social relations from a transformative perspective, particularly useful for studies in rural contexts. This formulation was a milestone, as it shifted the understanding of empowerment from mere resource access to a relational, dynamic, and situated process.

### Institutionalization and Measurement (2002–2005)

The entry of the concept into international policy frameworks is evident in the work of Malhotra et al. (2002), who developed a framework for measuring women’s empowerment in development programs in a report for the World Bank. This work was decisive in operationalizing the concept for use in impact assessments and logical frameworks.

In 2005, Kabeer advanced this discussion in her article Gender Equality and Women’s Empowerment: A Critical Analysis of the Third Millennium Development Goal, in which she criticized the instrumental and superficial use of the concept in the Millennium Development Goals. Kabeer argued that empowerment should be understood as the process by which those denied the ability to choose acquire such capacity, integrating agency, resources, and achievements.

### Consolidation and Global Expansion (2010–2020)

From 2010 onward, the concept of rural women’s empowerment was consolidated as a key axis in both academic agendas and international policy frameworks. Its inclusion in the Sustainable Development Goals (SDGs)—particularly SDG 5—triggered sustained growth in rural-focused research, with a variety of approaches: economic, productive, educational, digital, and community empowerment.

During this phase, the concept underwent diversification and regionalization, incorporating local knowledge and intersecting with issues such as poverty, food security, leadership, and sustainability.

Regarding the contribution of this research, the study highlights the comprehensive systematization of a thematically mature yet bibliometrically fragmented phenomenon. By applying scientometric techniques and conceptual mapping, it offers a structured and global perspective on rural women’s empowerment, guiding future research toward identified gaps.

Among the limitations, the exclusive use of the Scopus database is noted, which may introduce coverage bias by excluding literature indexed in other databases or published in other languages. In addition, the analysis was based on metadata only, without access to the full content of the documents, which limits in-depth interpretation of the theoretical and methodological frameworks employed in each study.

For future research, it is proposed to expand the search to other core databases such as Web of Science or RedALyC to incorporate regional literature and multilingual sources. It is also recommended to complement bibliometric analysis with systematic reviews or qualitative mappings in order to delve deeper into the analytical frameworks used. Finally, the study encourages researchers to strengthen interinstitutional and international collaboration networks, with the aim of enriching interdisciplinary dialogue and advancing toward more integrated strategies for empowerment in rural territories.

## Conclusions

The bibliometric review conducted in this study has provided a comprehensive understanding of the scientific production related to rural women’s empowerment, including trends, key actors, and structural dynamics in current research on the topic. This body of literature shows, first and foremost, a sustained increase in publications since 2015, largely driven by the inclusion of rural women’s empowerment in the Sustainable Development Goals (SDGs) and the increased visibility of gender inequalities following the COVID-19 pandemic. This growth signals a paradigm shift, wherein rural women are no longer seen merely as beneficiaries of development aid programs but are increasingly recognized as agents of social and economic change.

The scientific contribution of authors in this field aligns with Lotka’s Law, showing a strong concentration of publications among a small group of authors, while a large percentage have published only a single article. Noteworthy contributions come from Mudhara M and Aziz N, whose publications have generated significant impact shortly after being released.

Bradford’s Law identifies journals such as Environment, Development and Sustainability and World Development as part of the core zone of specialization in the dissemination of knowledge on this topic, highlighting their relevance as essential sources of reference.

The analysis of collaboration networks revealed considerable fragmentation and limited cooperation among authors and institutions, underscoring the need to establish interdisciplinary networks and foster international collaborations in order to approach rural women’s empowerment from a more systemic perspective.

Most of the research funding originates from dispersed sources, with a few prominent contributors, such as the Bill and Melinda Gates Foundation and the Ministry of Science and Technology of China, emphasizing the opportunity to consolidate more coherent and coordinated support structures.

The thematic analysis and semantic maps revealed the plural and multidimensional nature of the concept of empowerment, encompassing economic, social, and political aspects. Terms such as agency, gender equality, and rural development emerged as foundational pillars, while others—such as microfinance and leadership—represent more specific areas with strong potential for integration.

The historical roots of the concept, from the seminal works of Boserup (1970) and Freire (1970) to the contributions of Kabeer (2005), reflect a progression from critical approaches to practical frameworks applied in global development policies.

In this regard, the present study offers a thorough overview of the current state of rural women’s empowerment, highlighting its growing relevance in academic research and its clear move toward interdisciplinarity. Nevertheless, several challenges remain, including thematic fragmentation and limited collaboration among researchers.

Despite these challenges, future research directions should focus on fostering scientific networks, engaging in debates on key theoretical and methodological issues, and examining the role of funding sources in supporting transformative research that contributes to achieving gender equity in rural settings.

### Ethical statement

This study is a bibliometric review based exclusively on secondary data obtained from the Scopus database. All data used were bibliographic metadata from previously published scientific documents. No human participants, animals, or identifiable personal data were involved in the research. Therefore, ethical approval and informed consent were not required. The study complies with the ethical standards for research integrity, transparency, and responsible data use.

### Underlying data

Zenodo: Metadata collection database from Scopus, processed data, and results from the project Empowerment of Rural Women: A Bibliometric Review. https://doi.org/10.5281/zenodo.15708672 ( Vela Meléndez et al., 2025).

The dataset includes: The Scopus metadata collection, processed data in Bibliometrix y figures generated based on the dataset.

The data are available under the terms of the Creative Commons Attribution 4.0 International License (CC-BY 4.0). Raw data files are provided in CSV format.
