# Supplementary material for: Empowerment of rural women. A bibliometric review
Source: F1000Res. 2025 Jul 15;14:693. [Version 1] doi: 10.12688/f1000research.166893.1 (PMC12749559; doi:10.12688/f1000research.166893.1)
Supplement: Peer review history

# Reviewer report: Reviewer response for version 1

**DOI:** 10.5256/f1000research.183948.r433043

**Published:** 2025-12-29

**Authors:** Dailén Noa Guerra

**Affiliations:** 1 Universidad Ciego de Ávila Máximo Gómez Báez, Ciego de Ávila, Cuba

It is suggested that you review the introduction for a better understanding; there are inconsistencies in the writing, loose ideas without logical connectors, and a lack of independent criteria. Some references do not have all the necessary information for access and verification.

Is the work clearly and accurately presented and does it cite the current literature?

Yes

If applicable, is the statistical analysis and its interpretation appropriate?

Yes

Are all the source data underlying the results available to ensure full reproducibility?

Yes

Is the study design appropriate and is the work technically sound?

Yes

Are the conclusions drawn adequately supported by the results?

Yes

Are sufficient details of methods and analysis provided to allow replication by others?

Yes

Reviewer Expertise:

local development, rural development, rural tourism, tourism modalities developed in rural areas, role of public administration, governance, tourism potential in rural areas, planning and management in rural areas

I confirm that I have read this submission and believe that I have an appropriate level of expertise to confirm that it is of an acceptable scientific standard, however I have significant reservations, as outlined above.

# Reviewer report: Reviewer response for version 1

**DOI:** 10.5256/f1000research.183948.r433038

**Published:** 2025-12-24

**Authors:** Sukhdeep Kaur Mann

**Affiliations:** 1 Punjab Agricultural University, Ludhiana, Punjab, India

The paper offers a thorough bibliometric analysis of rural women's empowerment, succinctly describing authorship patterns, theme structures, publication trends, and conceptual foundations. The study design is appropriate and technically sound; however, full reproducibility is limited because Scopus data cannot be publicly shared. Overall, the conclusions are well supported by the results. The discussion effectively links empirical findings to theoretical frameworks and historical developments.

Is the work clearly and accurately presented and does it cite the current literature?

Yes

If applicable, is the statistical analysis and its interpretation appropriate?

Yes

Are all the source data underlying the results available to ensure full reproducibility?

No

Is the study design appropriate and is the work technically sound?

Yes

Are the conclusions drawn adequately supported by the results?

Yes

Are sufficient details of methods and analysis provided to allow replication by others?

Yes

Reviewer Expertise:

Information & Communication Technology, Women Empowerment, Diffusion and Adoption of technologies

I confirm that I have read this submission and believe that I have an appropriate level of expertise to confirm that it is of an acceptable scientific standard.

# Reviewer report: Reviewer response for version 1

**DOI:** 10.5256/f1000research.183948.r399792

**Published:** 2025-09-16

**Authors:** Mayuri Bora

**Affiliations:** 1 Assam Agricultural University, Jorhat, Assam, India

Yes, the study has been clearly and accurately presented including the abstract, introduction, review of literature, methodology, Results and discussion and conclusions.

 In terms of citing current literature, the study refers to works published in 2010, 2017, 2018, and 2024, while also considering the post-publication utility of articles in 2025. However, there remains scope for analyzing the trends in thematic areas covered across the years.

 The research design is appropriate and technically sound, adhering to the standard dimensions of a research article.

 The study employs a quantitative approach, using scientometric and bibliometric methods. The software and statistical analyses used are clearly described, ensuring replicability for future researchers. The source data are also reproducible.

 The conclusions are adequately supported by the data. Nevertheless, the study could be strengthened by offering additional recommendations for future research directions.

Is the work clearly and accurately presented and does it cite the current literature?

Yes

If applicable, is the statistical analysis and its interpretation appropriate?

I cannot comment. A qualified statistician is required.

Are all the source data underlying the results available to ensure full reproducibility?

Yes

Is the study design appropriate and is the work technically sound?

Yes

Are the conclusions drawn adequately supported by the results?

Yes

Are sufficient details of methods and analysis provided to allow replication by others?

Yes

Reviewer Expertise:

Women in agriculture, Empowerment of rural women, rural and sustainable tourism

I confirm that I have read this submission and believe that I have an appropriate level of expertise to confirm that it is of an acceptable scientific standard, however I have significant reservations, as outlined above.
